# Ho_3_Fe_5_O_12_ nanoparticles immobilized on FPS for production of a biopolymer from CO_2_ and limonene epoxide

**DOI:** 10.1039/d4ra05285d

**Published:** 2024-11-22

**Authors:** Fatemeh Shafiezadeh, Ali Javid, Rahele Zhiani, Sadegh Allameh

**Affiliations:** a Department of Chemistry, Mashhad Branch, Islamic Azad University Mashhad Iran Alijavids@yahoo.com; b Department of Chemistry, Neyshabur Branch, Islamic Azad University Neyshabur Iran; c New Materials Technology and Processing Research Centre, Neyshabur Branch, Islamic Azad University Neyshabur Iran

## Abstract

Herein, we present the synthesis of nanocatalysts with a large surface area. This was achieved through an interaction involving tetraethyl orthosilicate (TEOS) and tripolyphosphate (TPP), followed by the coupling of a ruthenium acetate complex with the click-transformed ligand of filamentous phosphosilicate (FPS). As a result, Ho_3_Fe_5_O_12_ nanoparticles were uniformly distributed without aggregation over FPS, forming Ho_3_Fe_5_O_12_@FPS. This substance was subsequently employed as a green nanocatalyst for the synthesis of cyclic carbonate from carbon dioxide and limonene epoxide whilst adhering to eco-friendly conditions. In the next step, we attempted to synthesize a polymer from synthesized natural cyclic carbonate. The incorporation of threadlike FPS divisions increased the ability to adsorb and aided the retrieval of the adsorbent without notably diminishing its effectiveness. The formed products were easily separated from the eco-friendly medium, and the catalyst was reused many times without a noticeable decrease in its activity and specificity.

## Abstract 2

Ho_3_Fe_5_O_12_@FPS was synthesized for the first time using an advanced and environmentally friendly method. Subsequently, Ho_3_Fe_5_O_12_@FPS was used as a long-lasting and reusable adsorbent for the reaction of carbon dioxide with limonene epoxide.

## Graphical abstract

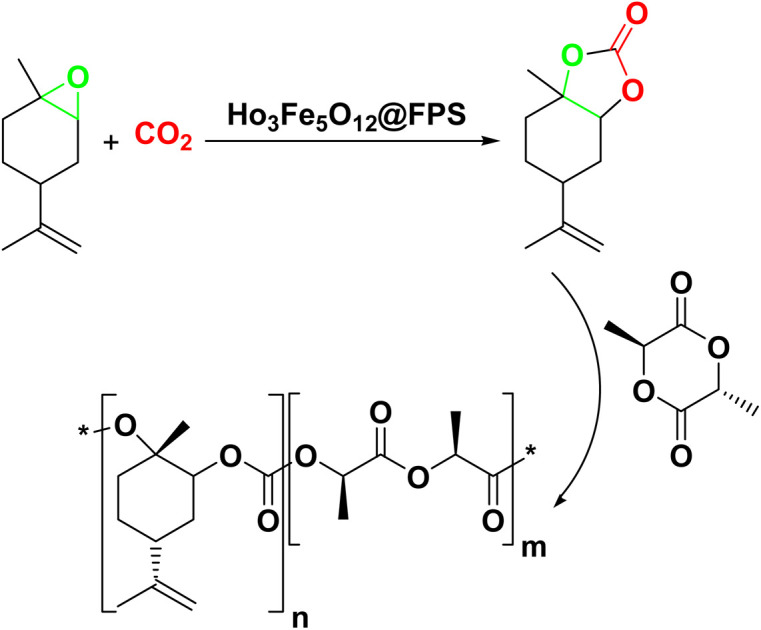

## Introduction

In this study, we created a fibrous nanostructure composed of phosphorus and silica. In the field of nanocatalysis, mesoporous materials such as FPS are often chosen for industrial applications.^1^ The large surface area of this material is attributed to the existence of dendrimeric P and Si fibers and their distinct passageways, making this FPS a unique material.^2^ The use of a surfactant in FPS resulted in a new morphology of the mesoporous silica material with dioxide strands and dendrimeric phosphate silicon.^3–5^ This could be investigated as an auxiliary substance in absorption and catalytic procedures. FPS provides a high surface area, thus enhancing the conversion of reactants into products.^6–10^

The increase in carbon dioxide concentrations in the atmosphere, attributed to anthropogenic economic expansion, has resulted in significant environmental implications. However, carbon dioxide can serve as an economical and abundant material for the synthesis of diverse pivotal formulated constituents, encompassing oxazolidinones,^11,12^ carboxylic acids,^13^ and cyclic carbonates.^14^ Additionally, carbon dioxide is susceptible to efficient reduction to yield precious synthesized feedstocks such as formaldehyde,^15^ formic acid,^16^ and methanol.^17^ A remarkable utilization of carbon dioxide is the production of aldehyde *via* the *N*-formylation of amines and CO_2_,^18^ as originally suggested by other scientists employing nanocatalysts.^19–23^

Nonetheless, these thermocatalytic methods generally necessitate high pressure, an external energy source, and either uniform phase catalysts or costly rare-earth elements. The employment of ionic liquids as catalysts has facilitated the successful cycloaddition of natural epoxide with CO_2_ at room temperature.^24^ Furthermore, a reliable approach encompassing the photocatalytic methylation and formylation of amines with CO_2_ under mild conditions holds substantial potential.^25^ Prajapati and his team engineered a CuO–BiVO_4_ photocatalyst for the cycloaddition of natural epoxide with carbon dioxide. In addition, Malik and his group realized a throughput of 97% for the cycloaddition of epoxide utilizing a zinc phthalocyanine–C_3_N_4_ composite photoreactive system. However, additional research is required to understand the processes that underpin these light-activated catalysis.^26^

In the quest for sustainable solutions, bio-based polymers have taken center stage as environmentally friendly alternatives to traditional plastics.^27^ One such promising bio-based polymer is poly(limonene carbonate) (PLimC), recognized for its potential in various applications.^28^ PLimC was pioneered by Coates and his team utilizing a μ-bis-diiminato zinc complex catalyst for the polymerization of limonene oxide.^29^ PLimC stands out as it is derived from *trans*-limonene oxide, which can be obtained by oxidizing limonene, a primary component of citrus oil.^30^ This monomer is not only sustainable but also abundant, with an impressive 57 000 tons of citrus oil being collected annually from orange juice production.^31^ The utilization of such renewable resources not only underscores PLimC's sustainable nature but also provides a valuable use for by-products from citrus juice processing.^32^ Additionally, PLimC boasts several attractive properties including transparency, high glass transition temperature, and biodegradability, making it suitable for a range of applications such as packaging, coatings, and medical devices.^33^ By harnessing the natural abundance of limonene and advanced polymerization techniques, PLimC can significantly reduce dependency on petrochemicals-derived plastics.^34^ The development and utilization of poly(limonene carbonate) (PLimC) aligns seamlessly with the global push towards sustainability.^35^ Its origins from renewable sources like citrus oil, combined with its promising physical properties, make PLimC an attractive candidate for replacing conventional plastics in numerous sectors.^36^ As research and interest continue to grow, PLimC could well become a cornerstone in the sustainable materials landscape.

**Scheme 1:**
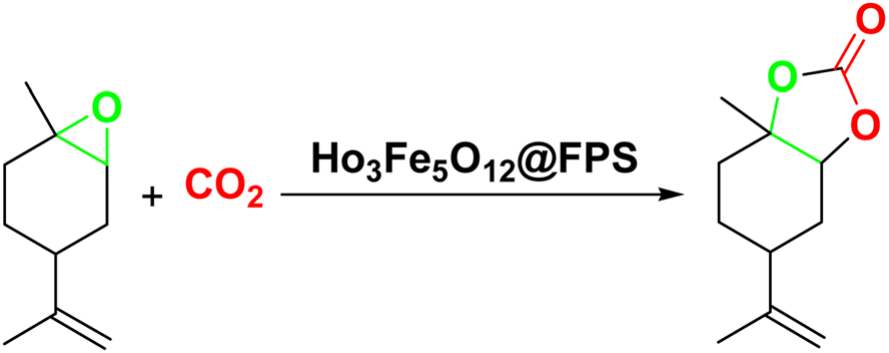
Production of cyclic carbonate from carbon dioxide and limonene epoxide.

**Scheme 2:**
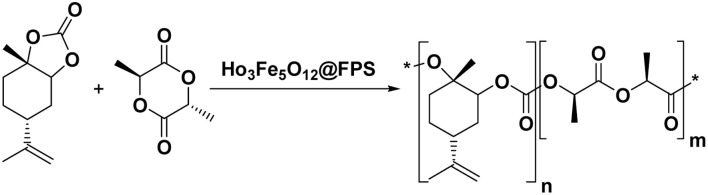
Ho_3_Fe_5_O_12_@FPS-catalyzed synthesis of the polymer.

Magnetic nanoparticles have recently garnered significant interest from researchers due to their appealing properties. These characteristics could be utilized for the effective extraction of catalytic substances from different chemical processes by introducing an exterior field, enabling their later retrieval. The simplicity of separation using a magnetic stimulus, together with a high surface-to-volume ratio, endows magnetic nanoparticles with the capability to cleanse impure water. Magnetic nanoparticles are able to exhibit adsorption behavior in the operation under study and serve as magnetically-guided carriers of absorbent substances situated on their exterior layer. Orthoferrites of rare-earth elements RFeO_3_ (R = La–Lu) are a type of magnetic substances that have garnered significant interest owing to their reliable functionality and robust durability.^37^ Amidst these orthoferrites, holmium orthoferrite is particularly notable as it exhibits properties of a visible light photocatalyst,^38^ a possible ambient-heat level multifunctional magnetic material,^39^ and an antiferromagnet with the important effect of spin realignment.^40^ However, the activity of holmium orthoferrite in antibiotic adsorption processes remains unexplored despite the inherent potential of its magnetic performance. Conversely, magnetic oxide composites have the prospect of exhibiting the sought-after magnetic features, a large surface area, numerous active sites, and an excellent ability to accumulate substances on the surface.^41^ In the past, various magnetic nanocomposites have been utilized to extract or absorb biological and non-biological pollutants from water-based solutions.^42,43^ Another category of magnetic materials is ferrite garnets, which possess non-equivalent and antiferromagnetically linked spin substructures.^44^ Within this group, ferromagnetic rare earth iron garnets have garnered considerable interest owing to their unique properties, magneto-caloric characteristics,^45^ including magneto-optical,^46^ and magneto-dielectric.^47^ In this study, Ho_3_Fe_5_O_12_@FPS was synthesized for the first time using an advanced and environment-friendly method. Subsequently, Ho_3_Fe_5_O_12_@FPS was used as a long-lasting and reusable adsorbent for the reaction of carbon dioxide with limonene epoxide (Scheme 1). In the next step, we tried to synthesize the polymer from the synthesized natural cyclic carbonate (Scheme 2).

## Experimental section

### Creation of FPS

2.89 g of tetraethyl orthosilicate and 3.96 g of tripolyphosphate were combined in a mixture of cyclohexane (21 mL) and 1-pentanol (1.6 mL). A stirred mixture of CPB (1.4 g) and urea (0.8 g) in 32 mL of water was subsequently introduced to the initial solution. The final mixture was stirred continuously for 1.2 hours at room temperature before being moved to a Teflon-lined hydrothermal autoclave and the temperature was maintained at 120 °C for a duration of 5 hours.

### Creation of Ho_3_Fe_5_O_12_@FPS using microwave technique

Newly prepared FPS (50 mg), Ho(NO_3_)_3_·5H_2_O (0.7 mmol), and Fe(NO_3_)_3_·9H_2_O (5 mmol) were mixed with a PEG aqueous solution. The mixture was continuously stirred for 1 hour at room temperature before being heated at 115 °C for 2.5 hours to trigger the chemical process. Once the reaction was finished, the mixture was cooled to room temperature. The Ho_3_Fe_5_O_12_@FPS core–shell microspheres were then separated using strong magnetic suction, rinsed with deionized water and acetone, and left to dry in a drying oven at 40 °C overnight. The material was finally calcined in air at 480 °C for 3.5 hours.

### A heterogenous method for cycloaddition

In a typical reaction, a mixture of 8 milligrams of Ho_3_Fe_5_O_12_@FPS, together with limonene epoxide (0.5 millimole) and 10 millilitres of CH_3_CN, were inserted into a 50 millilitre reaction vessel. The evaluation of the catalytic activity was done under magnetically-induced agitation parameters at a pressure of 2.5 MPa of CO_2_ for a duration of 15 minutes. The resulting mixture was heated up to 70 °C.

### A heterogeneous synthesis method for the polymer

Cyclic carbonate (0.1 mmol), Ho_3_Fe_5_O_12_@FPS (6 mg), lactide (2.0 mmol), CH_3_CN (10 mL), and NaOAc (2.0 milligrams) were introduced into a 50 milliliter reaction vessel, which was then sealed. The resulting mixture was heated up to 60 °C. The progress of the reaction was monitored using thin-layer chromatography (TLC). Following completion, ethanol was introduced into the blend and subsequent filtration was carried out to remove the catalyst. The solvent was subsequently extracted under reduced pressure, and the resulting product was purified through recrystallization using ethyl acetate/*n*-hexane.

## Results and discussion

**Scheme 3:**
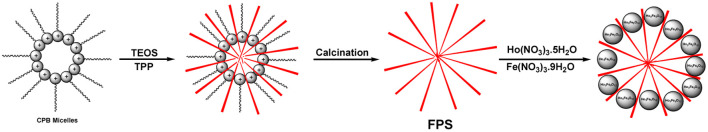
Scheme for the formulation of Ho_3_Fe_5_O_12_@FPS.

The process for preparing Ho_3_Fe_5_O_12_@FPS is outlined in Scheme 3. Nano-FPS was prepared using the microemulsion technique, with TPP and TEOS serving as the sources of P and Si and cetylpyridinium bromide (CPB) acting as a template in a mixture of water, cyclohexane, and pentanol. It is plausible that TPP and TEOS were hydrolyzed by urea, and the negatively-charged phosphate and silicate molecules filled the available space in the self-assembled template, thus surrounding it. The structure of these nanofibers includes FPS nanocomposite as the support material. FPS was then mixed with Fe(NO_3_)_3_·9H_2_O and Ho(NO_3_)_3_·5H_2_O to prepare Ho_3_Fe_5_O_12_@FPS. Furthermore, FPS acted as a center for aggregation, promoting the growth of the Ho_3_Fe_5_O_12_ nanocomposite on the outer layer of FPS.

**Fig. 1:**
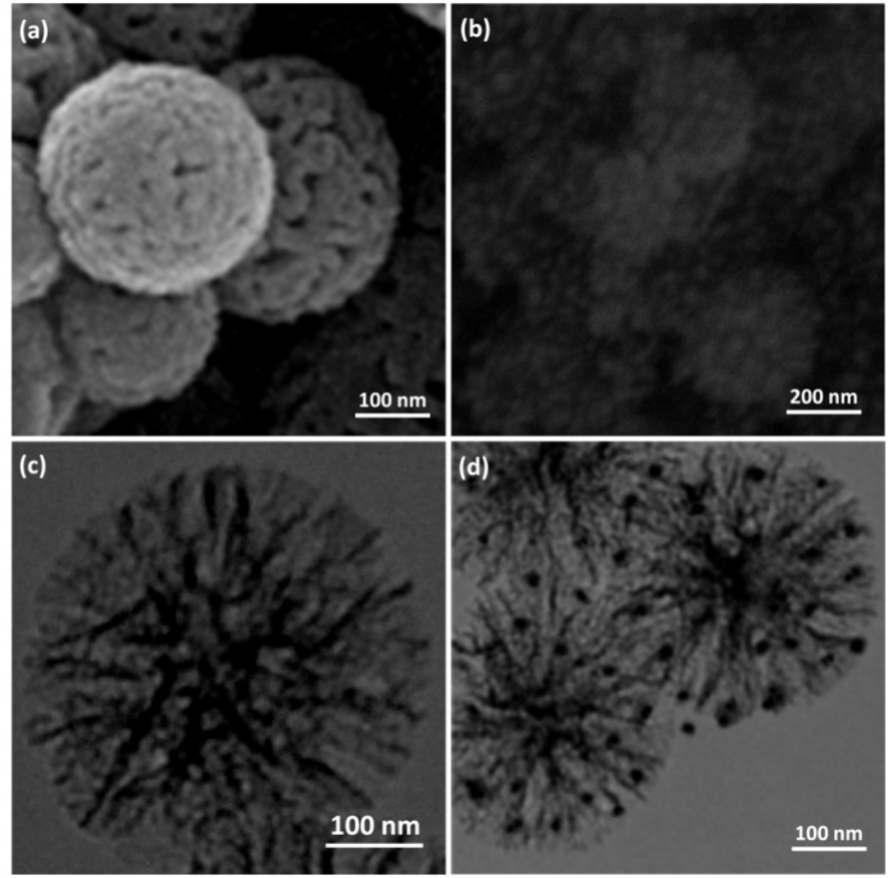
FESEM illustrations of (a) FPS and (b) Ho_3_Fe_5_O_12_@FPS; (c) TEM illustrations of FPS and (d) Ho_3_Fe_5_O_12_@FPS.

**Fig. 2:**
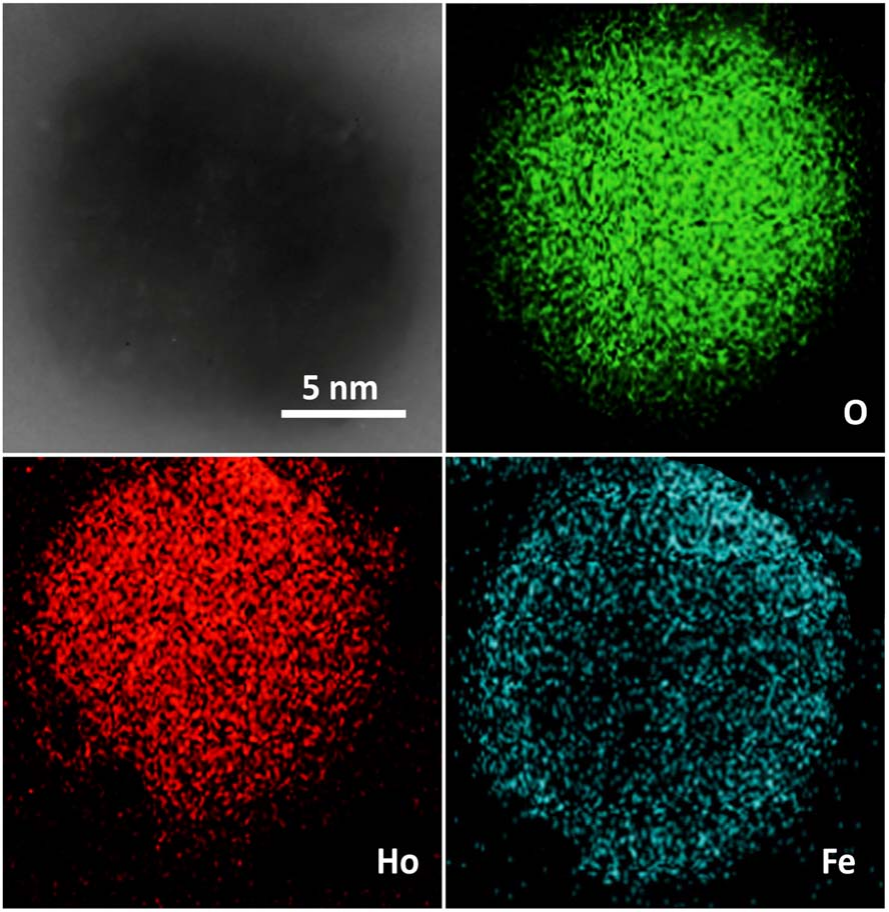
TEM mapping of Ho_3_Fe_5_O_12_ covering the overall state, Fe, O, and Ho.

In order to understand the underlying causes, we examined cells using SEM and TEM (Fig. 1 and 2). The Ho_3_Fe_5_O_12_@FPS MNPs possess three-dimensional dendrimeric fibers that form walls, facilitating easy access to the available surface. Fig. 1b reveals that Ho_3_Fe_5_O_12_ MNPs have a solid particle size of approximately 15–20 nm. This suggests the distributed of the formulated catalyst and a range of greater structures that might be attributed to the clustering or unification of nanoparticles. The MNPs seem to exhibit a spherical shape.

**Fig. 3:**
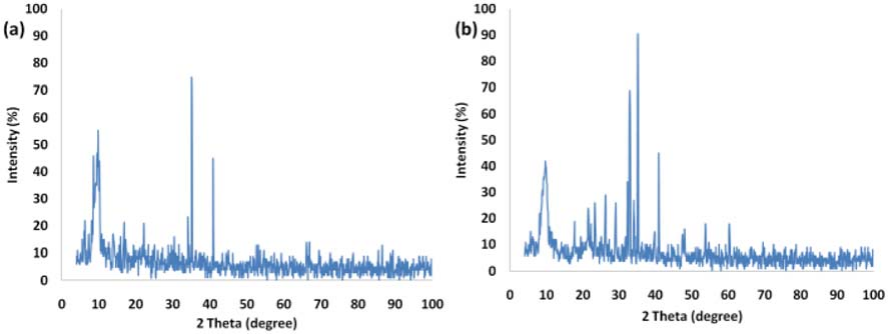
XRD arrangement of (a) FPS and (b) Ho_3_Fe_5_O_12_@FPS MNPs.

**Fig. 4:**
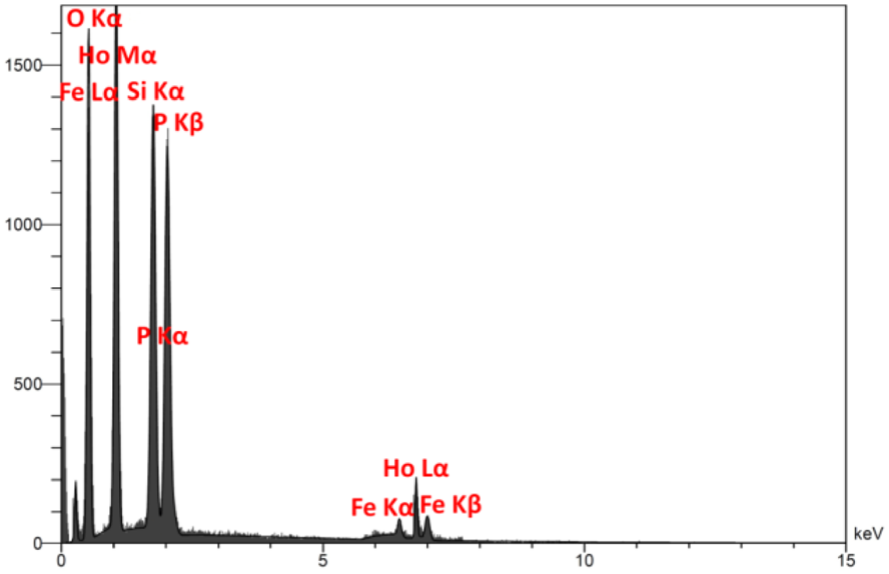
EDX analysis of Ho_3_Fe_5_O_12_@FPS MNPs.

Fig. 3 presents the XRD pattern of FPS and Ho_3_Fe_5_O_12_@FPS MNPs. The XRD pattern of FPS NPs exhibited several crystalline peaks that aligned with the results of similar studies.^48^ Additionally, new peak reflections of the Ho_3_Fe_5_O_12_ (JCPDS 01-074-1479) crystal were noted for Ho_3_Fe_5_O_12_@FPS MNPs, once more verifying the prosperous development of Ho_3_Fe_5_O_12_ particles on the FPS surface. The wide peak ranging from 20–30° was associated with amorphous silica. EDX was employed to identify the atoms in the Ho_3_Fe_5_O_12_@FPS MNPs. The presence of P, Si, Ho, Fe, and O indicated that Ho_3_Fe_5_O_12_@FPS was properly constructed (as shown in Fig. 4).

**Fig. 5:**
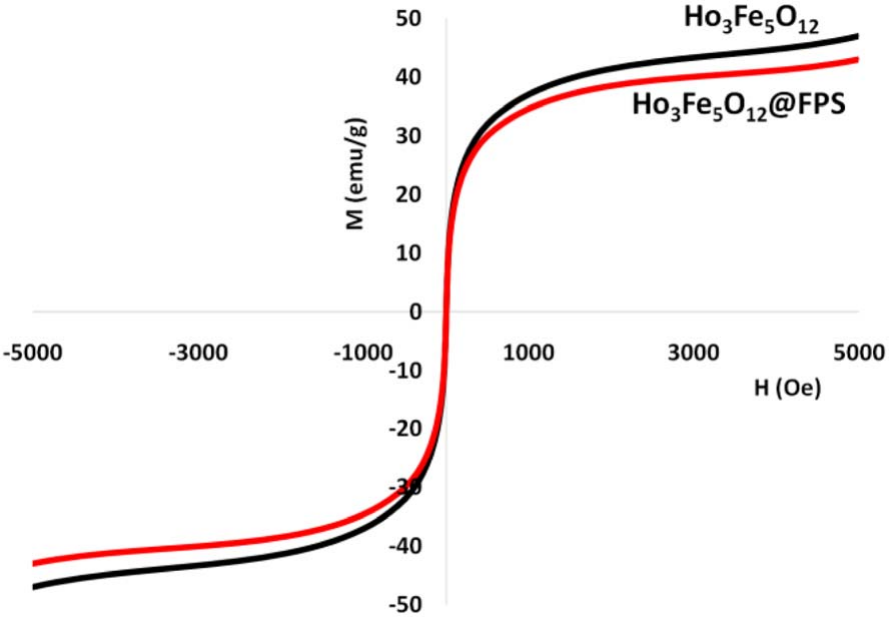
The magnetization curves of the nanocatalyst at room temperature.

**Fig. 6:**
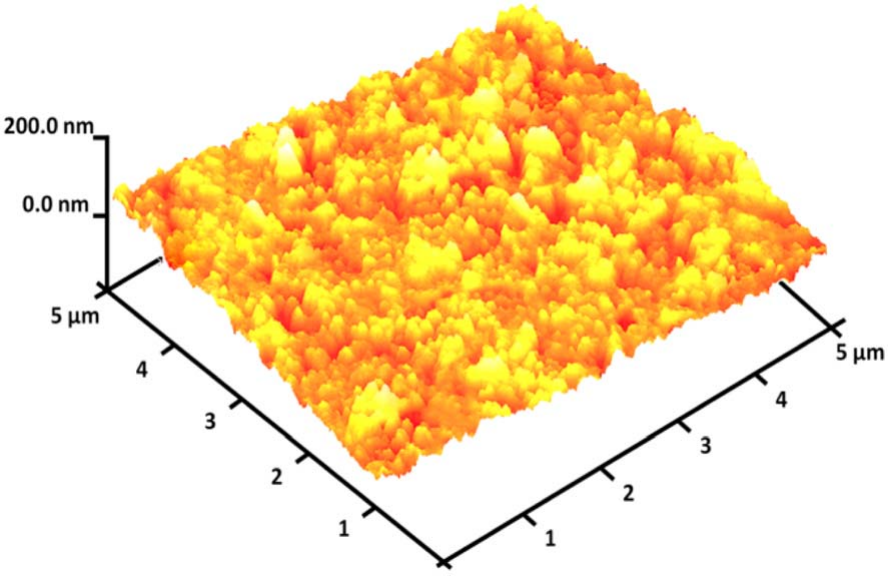
Three-dimensional AFM images of Ho_3_Fe_5_O_12_@FPS MNPs.

The magnetizable features of the MNPs were analyzed employing a vibrating sample magnetometer (VSM). The hysteresis loops of the resulting nanocomposite, recorded at 300 K, showed negligible remaining magnetic properties (Fig. 5). This finding indicated that the nanocomposite displayed paramagnetic properties. The magnetic measurements showed that the saturation magnetization values of pure Ho_3_Fe_5_O_12_ and Ho_3_Fe_5_O_12_@FPS MNPs were 46.9 and 18.4 emu g^−1^, respectively. The nanocomposite, possessing paramagnetic characteristics and a high degree of magnetization, could rapidly respond to an outside magnetic influence and promptly dissipate once the outside magnetic influence was withdrawn. These results demonstrated the nanocomposite's strong magnetic capabilities, suggesting its potential use in targeting and separation processes. The level of roughness of the Ho_3_Fe_5_O_12_@FPS nanoparticles was established using atomic force microscopy (AFM) analysis. As illustrated in Fig. 6, the taller areas (represented by a brighter yellowish-white color) boosted with a decrease in T/W, suggesting a rise in the level of roughness of the catalyst.

**Fig. 7:**
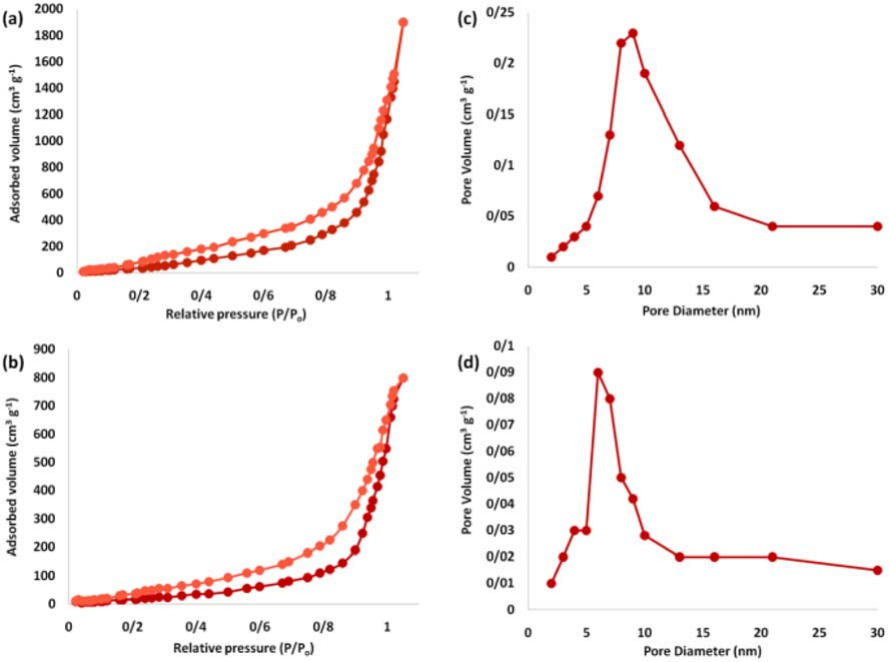
Adsorption–desorption isotherms of (a) FPS and (b) Ho_3_Fe_5_O_12_@FPS MNPs, and BJH pore size distributions of (c) FPS and (d) Ho_3_Fe_5_O_12_@FPS MNPs.

The nitrogen physisorption analysis revealed that the specific expanse of the BET for FPS and Ho_3_Fe_5_O_12_@FPS were 615 and 528 m^2^ g^−1^, respectively. The reduced expanse of Ho_3_Fe_5_O_12_@FPS compared to FPS could be attributed to the specification of Ho_3_Fe_5_O_12_. The nitrogen adsorption–desorption isotherms of the FPS-supported catalysts are depicted in Fig. 7. FPS demonstrated a type IV isotherm, with an H1-type hysteresis loop, suggesting the presence of mesopores. The associated pore size spectrum, predicted by the desorption branch of the nitrogen isotherm using the BJH approach, showed a narrow pore size peaking at 11 nm (Table 1). The substantial mesopore dimensions of FPS could accommodate Ho_3_Fe_5_O_12_, which has a relatively large molecular size.

**Table 1:** Architectural attributes of FPS and Ho_3_Fe_5_O_12_@FPS MNPs

Catalysts	*S*_BET_ (m^2^ g^−1^)	*V*_a_ (cm^3^ g^−1^)	*D*_BJH_ (nm)
FPS	615	3.2	12
Ho_3_Fe_5_O_12_@FPS	528	2.7	6

**Fig. 8:**
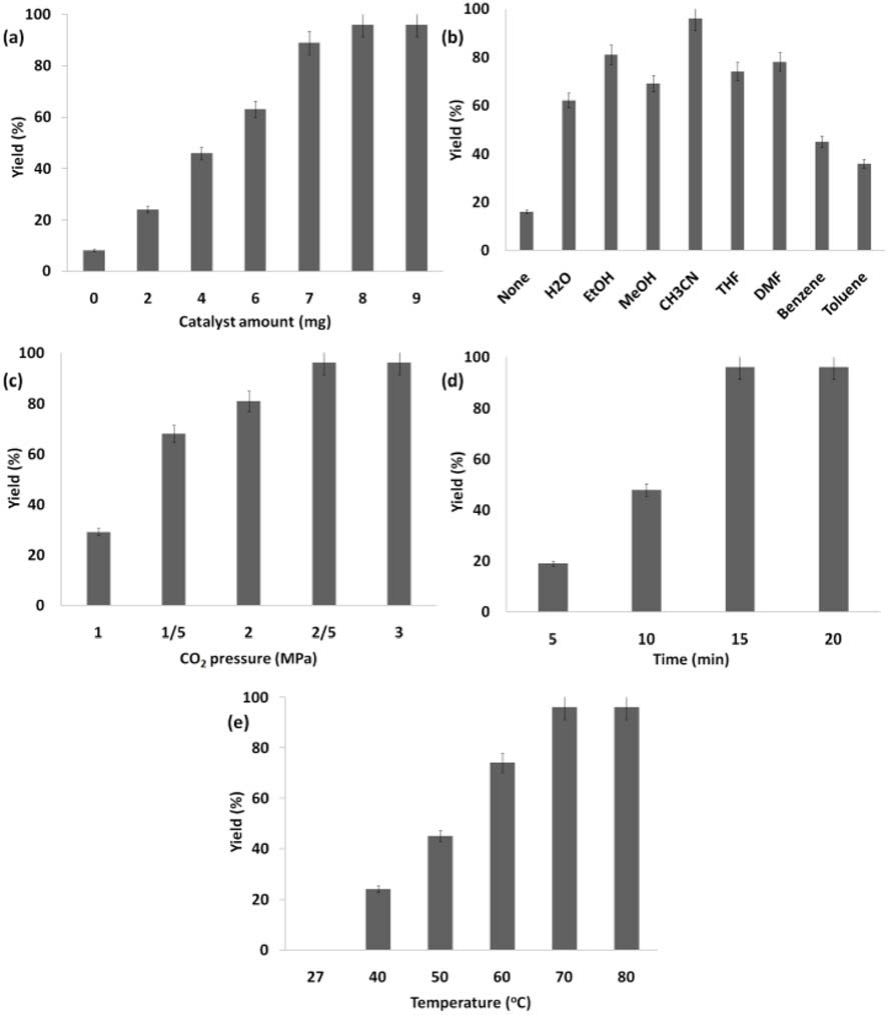
Effects of catalyst amount (a), solvents (b), the pressure of carbon dioxide (c), time (d) and temperature (e) on the catalyzed cyclic carbonate synthesis.

This research delved deeper into multiple reaction parameters pertaining to the synthesis of the product from carbon dioxide and limonene epoxide. These parameters encompass the quantity of the catalyst, the variety of π-conjugated nanoporous frameworks in the Ho_3_Fe_5_O_12_@FPS, and solvents. The findings suggested that the quantity of Ho_3_Fe_5_O_12_@FPS positively influenced the cycloaddition of natural epoxide with CO_2_ as the throughput increased up to 8 milligrams for Ho_3_Fe_5_O_12_@FPS (as depicted in Fig. 8a). CH_3_CN emerged as the quintessential solvating agent during the experimental process during the investigation process (as depicted in Fig. 8b). A throughput of 68% was realized when CO_2_ was subjected to a pressure of 1.5 MPa. Escalation in the force by circa 13% was noted upon the application of a singular partial force of 2.0 MPa, leading to a throughput of 81%. A peak yield of 96% was realized when the pressure was doubled, applying a total partial pressure of 2.5 MPa (Fig. 8c). However, any further increase in CO_2_ pressure resulted in a marked decrease in discrimination and transmutation due to the diminished steadiness and the ultimate fragmentation of the Ho_3_Fe_5_O_12_@FPS cluster. As shown in Fig. 8d, the starting materials were entirely converted into the respective product within a span of 15 minutes. The influence of temperature on the catalytic activity was examined. As indicated in Fig. 8e, no products were formed at room temperature. The production of cyclic carbonate increased with increasing temperature, achieving a 96% yield when 70 °C was utilized. Any increase in temperature did not change the product yield.

**Fig. 9:**
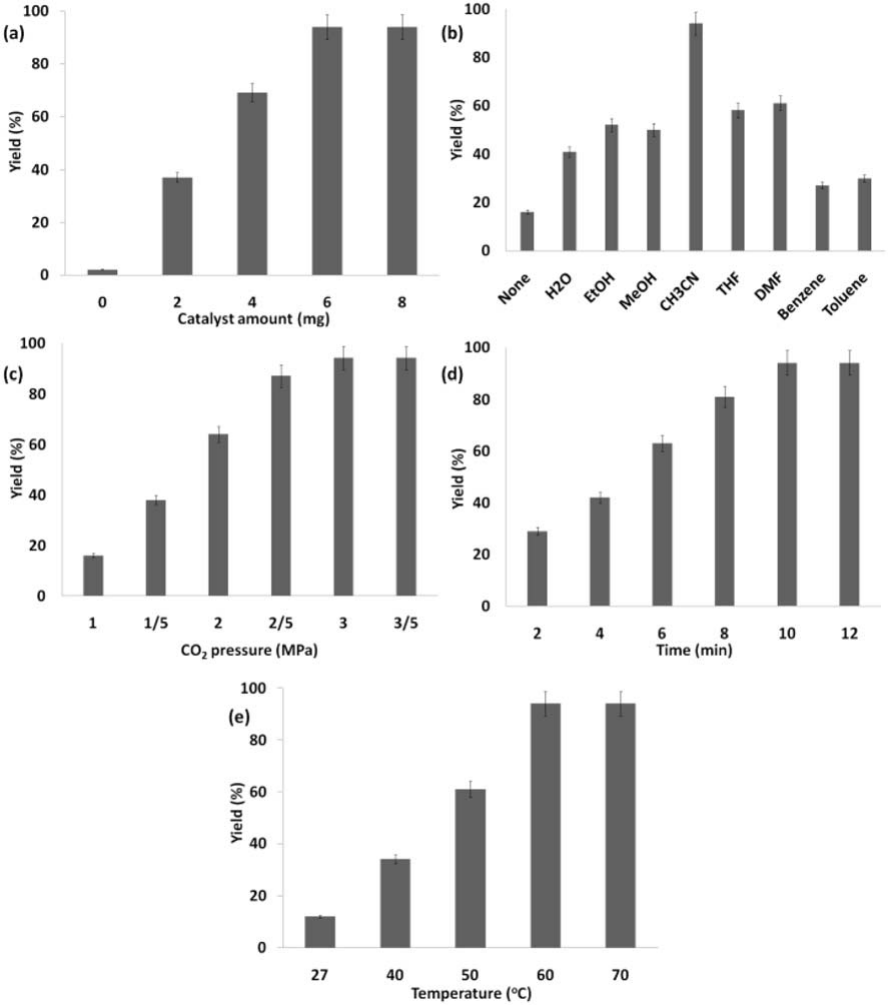
Synthesis of the polymer using Ho_3_Fe_5_O_12_@FPS NPs with various (a) catalyst amounts, (b) solvents, (c) pressures of carbon dioxide, (d) times, and (e) temperatures.

The progress of the reaction was monitored by GC for the shortest time with the addition of 6 mg of Ho_3_Fe_5_O_12_@FPS (Fig. 9a). No detectable by-products were obtained in any of the experiments, and the efficiency of the polymer was 94%. Poor results were obtained with other solvents, such as DMSO, EtOAc, THF, CH_3_CN, CHCl_3_, and CH_2_Cl_2_ (Fig. 9b), emphasizing the unique role of CH_3_CN in this reaction. The desired outcome was achieved with 94% efficiency using CH_3_CN as the solvent. The response was investigated under 3.0 MPa of CO_2_ (Fig. 9c). Under ideal situations, the highest synthesis efficiency of the polymer could be achieved in 10 minutes (Fig. 9d). The screening of the temperature showed that 60 °C was the optimal choice (Fig. 9e).

Under the most favorable reaction conditions, the substrate and two-component reactions were investigated (Table 2). It was found that several epoxides underwent this transformation to produce the intended outcomes with the highest efficiency. Different functional groups were well tolerated.

**Table 2:** Production of cyclic carbonates from CO_2_ and epoxides

Entry	Epoxides	Products	Yield (%)
1	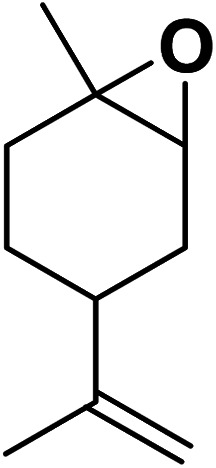	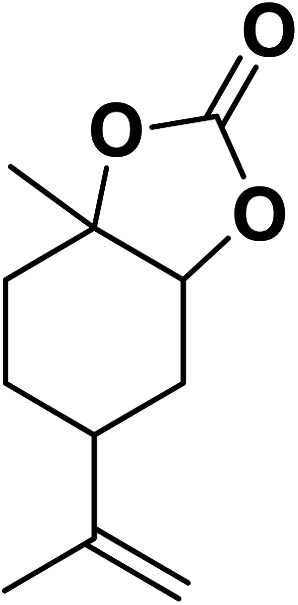	96
2	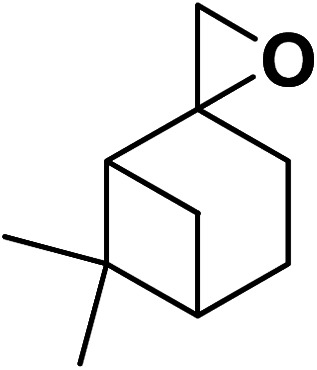	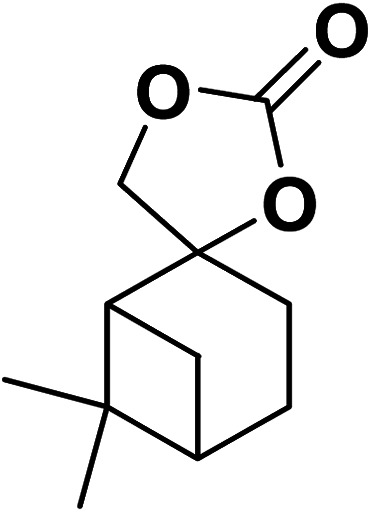	84
3	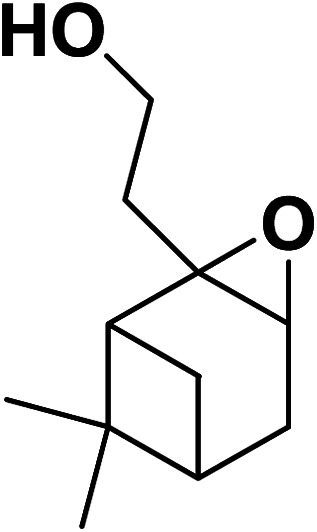	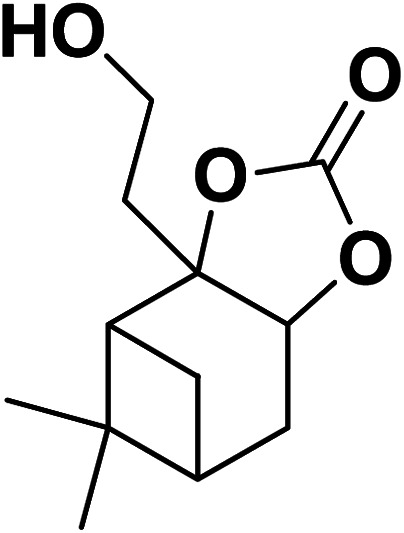	71
4	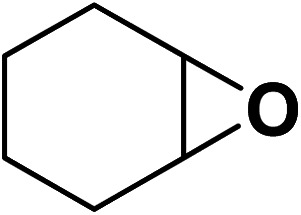	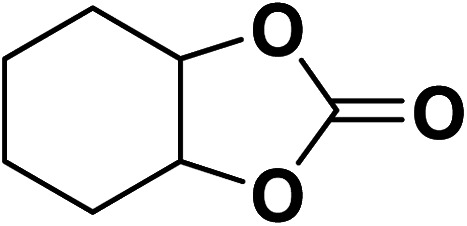	98

**Fig. 10:**
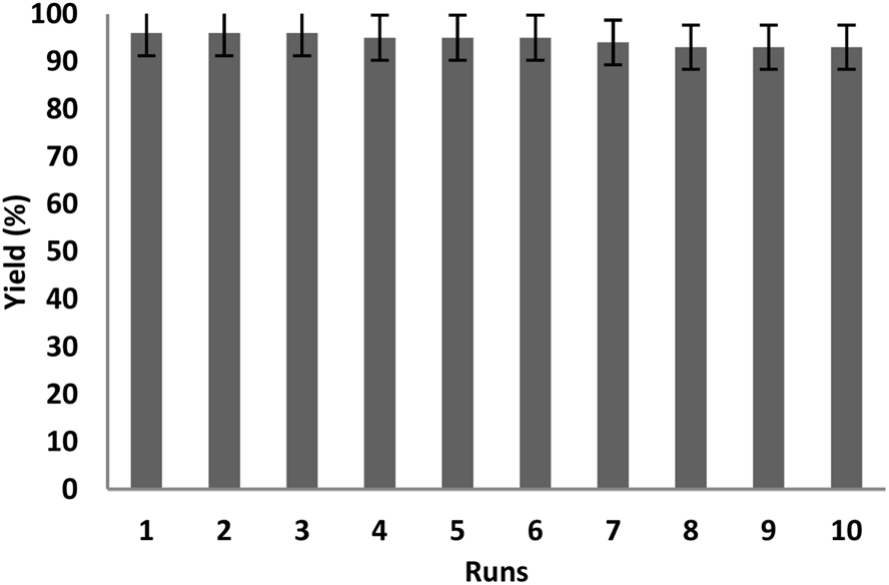
Catalyst performance in the re-synthesis of cyclic carbonate using Ho_3_Fe_5_O_12_@FPS.

To evaluate the recoverability of Ho_3_Fe_5_O_12_@FPS in the reaction, the catalyst was isolated after the completion of the reaction. Subsequently, it was rinsed with ethanol and water for utilization in the next reaction cycle. The results from ten iterations of reusing Ho_3_Fe_5_O_12_@FPS indicated that the nanocatalyst showed satisfactory recovery as there was no noticeable decrease in the efficiency until the fifth cycle. A slight decline in the efficiency of approximately 4% was observed in the tenth cycle (Fig. 10).

## Conclusions

We have developed a nanocatalyst that enables the precise tailoring of the energy and discrimination for the production of biopolymer from CO_2_ and limonene epoxide. We have introduced a family of Ho_3_Fe_5_O_12_@FPS exhibiting a distinct fibrous morphology. The Ho_3_Fe_5_O_12_@FPS exhibit noteworthy characteristics, such as a multitude of active sites, excellent mechanical and thermal resistance, and a vast external surface area. These remarkable properties allow for the potential utilization of NFs in various other reactions. The significant outer surface area, facilitated by the involvement of dendrimer wall-like plate nanoparticles and accompanying channels, renders Ho_3_Fe_5_O_12_@FPS a favourable choice for diverse scientific uses.

## Data availability

All information is written in the main text of the article and there is no additional information.

## Conflicts of interest

There are no conflicts to declare.
